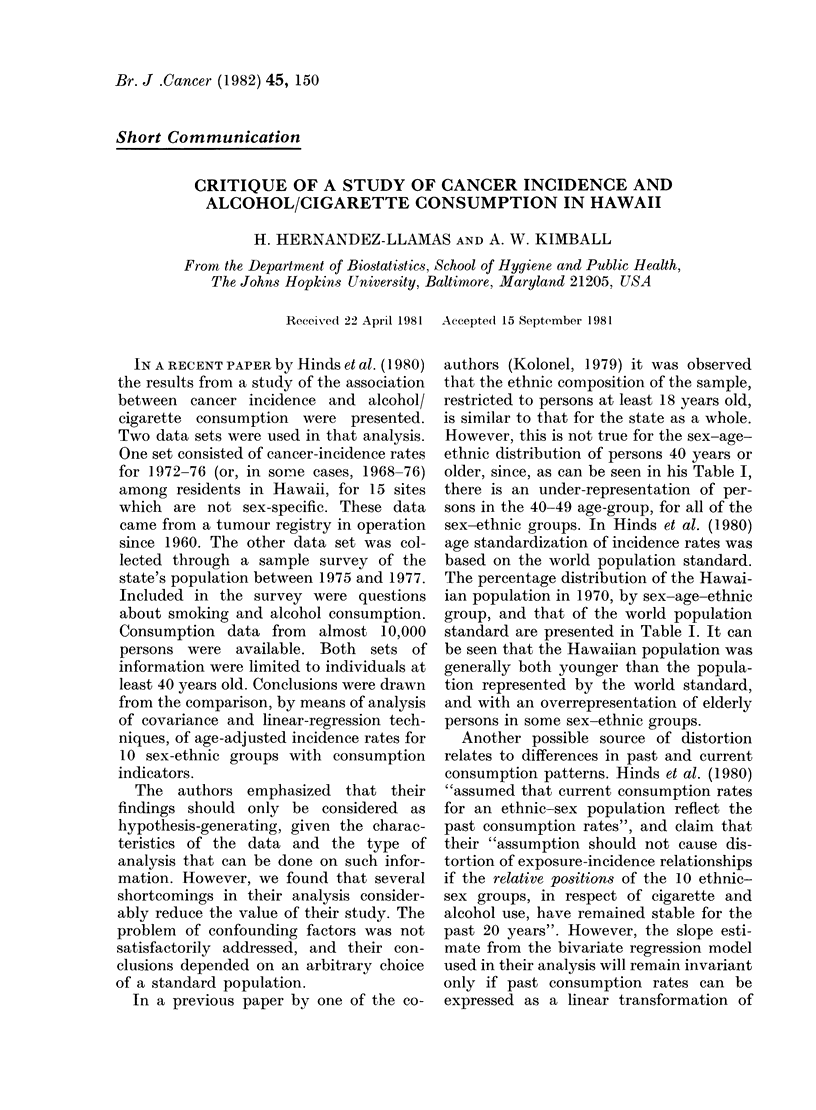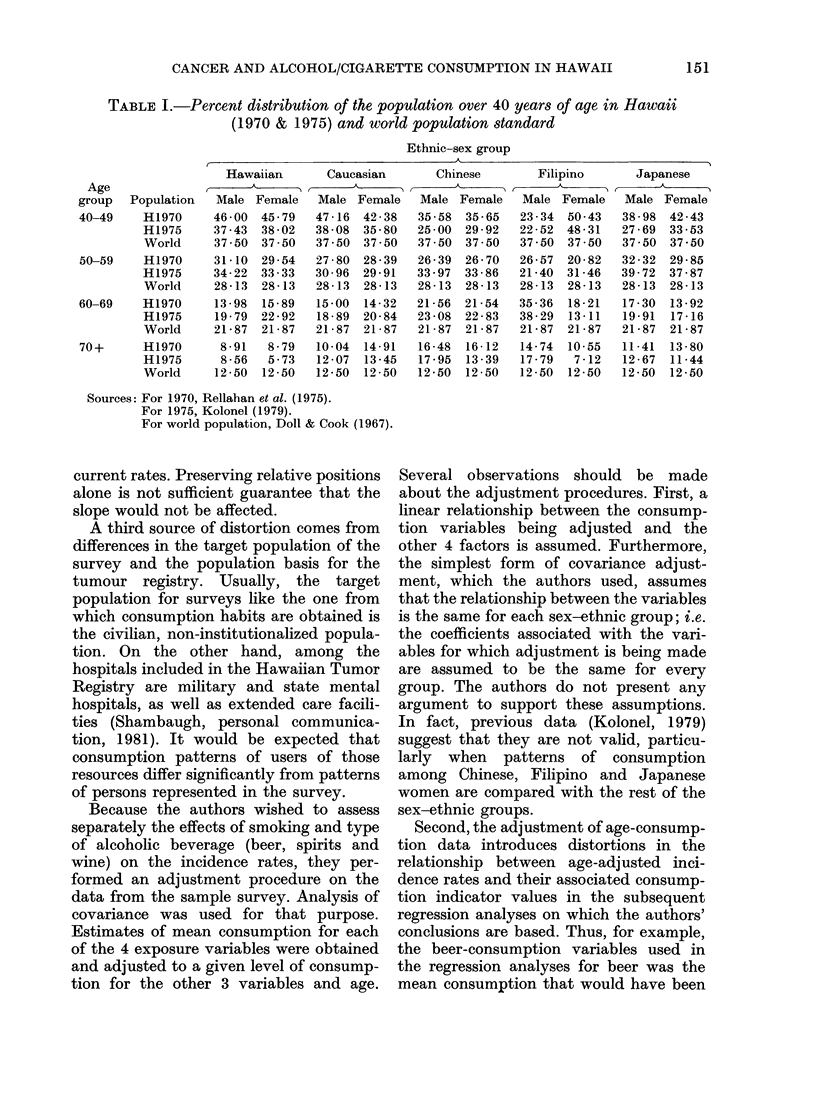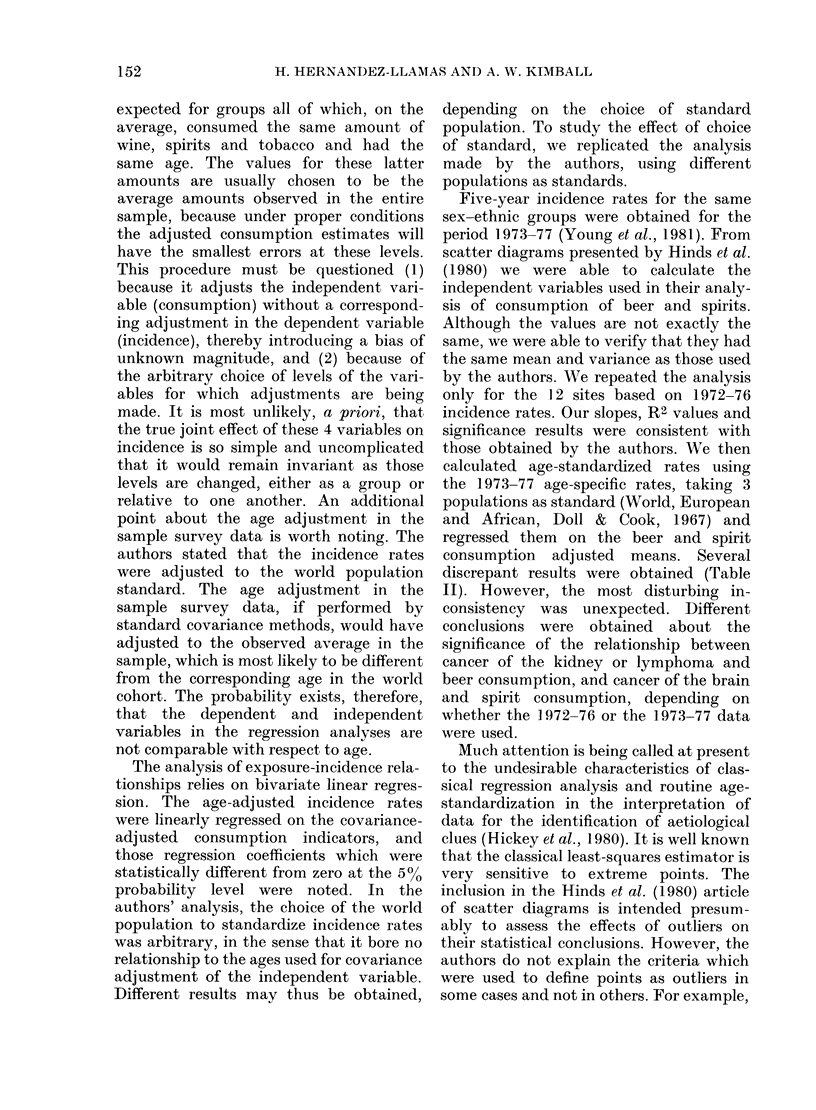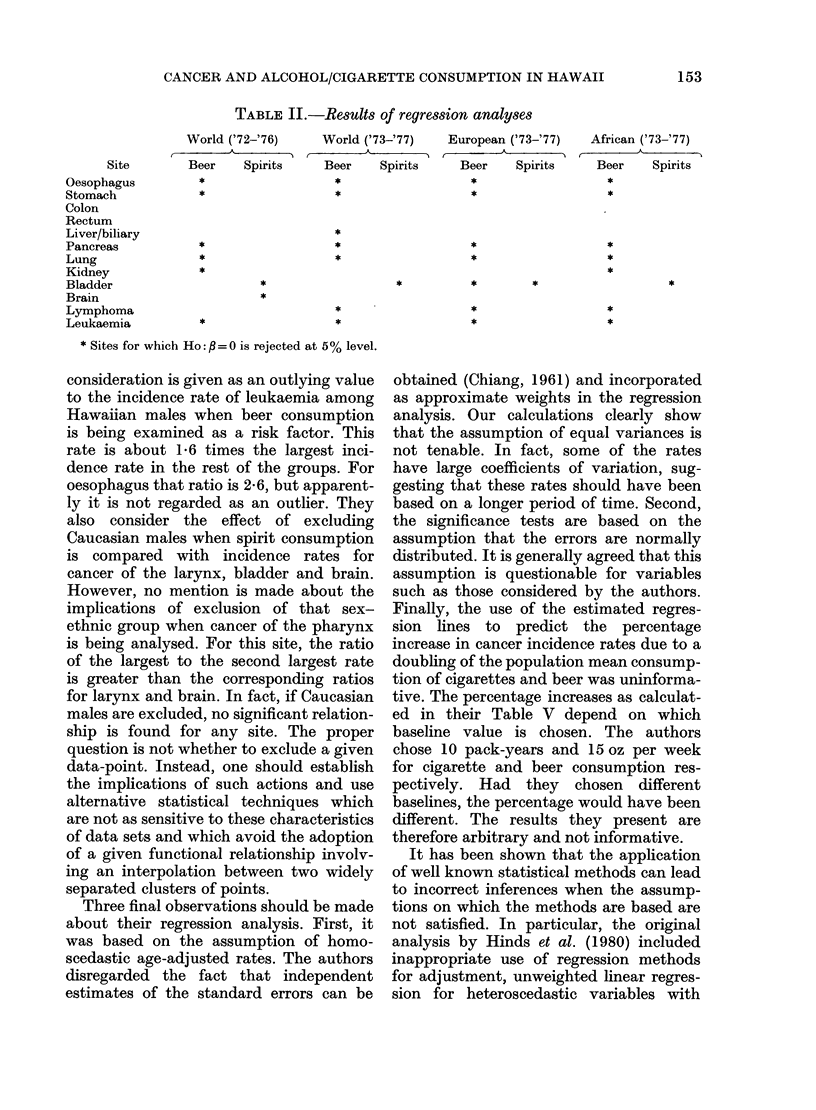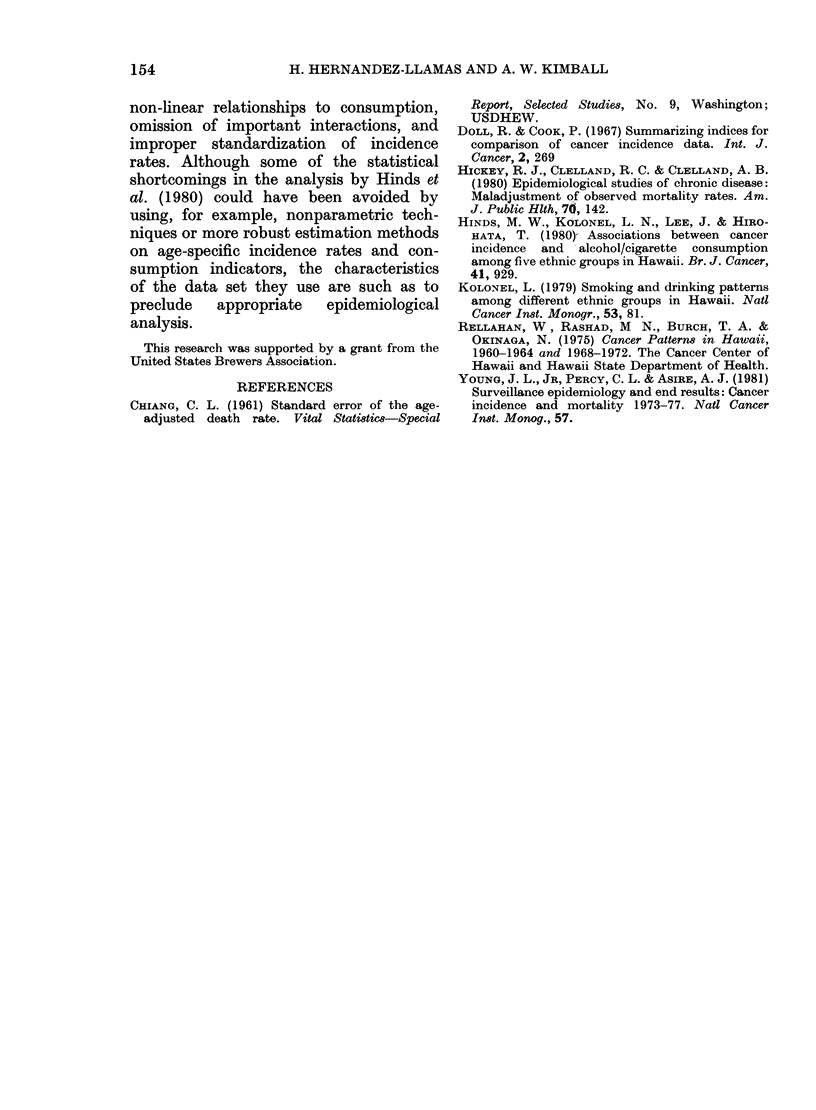# Critique of a study of cancer incidence and alcohol/cigarette consumption in Hawaii.

**DOI:** 10.1038/bjc.1982.20

**Published:** 1982-01

**Authors:** H. Hernandez-Llamas, A. W. Kimball


*Br. J .Cancer* (1982) **45**, 150

***Short Communication***

### CRITIQUE OF A STUDY OF CANCER INCIDENCE AND ALCOHOL/CIGARETTE CONSUMPTION IN HAWAII

H. HERNANDEZ-LLAMAS AND A. W. KIMBALL

*From the Department of Biostatistics, School of Hygiene and Public Health, The Johns Hopkins University, Baltimore, Maryland* 21205, *USA*

Received 22 April 1981 Accepted 15 September 1981

IN A RECENT PAPER by Hinds *et al.* (1980) the results from a study of the association between cancer incidence and alcohol/cigarette consumption were presented. Two data sets were used in that analysis. One set consisted of cancer-incidence rates for 1972–76 (or, in some cases, 1968–76) among residents in Hawaii, for 15 sites which are not sex-specific. These data came from a tumour registry in operation since 1960. The other data set was collected through a sample survey of the state's population between 1975 and 1977. Included in the survey were questions about smoking and alcohol consumption. Consumption data from almost 10,000 persons were available. Both sets of information were limited to individuals at least 40 years old. Conclusions were drawn from the comparison, by means of analysis of covariance and linear-regression techniques, of age-adjusted incidence rates for 10 sex-ethnic groups with consumption indicators.

The authors emphasized that their findings should only be considered as hypothesis-generating, given the characteristics of the data and the type of analysis that can be done on such information. However, we found that several shortcomings in their analysis considerably reduce the value of their study. The problem of confounding factors was not satisfactorily addressed, and their conclusions depended on an arbitrary choice of a standard population.

In a previous paper by one of the co-authors (Kolonel, 1979) it was observed that the ethnic composition of the sample, restricted to persons at least 18 years old, is similar to that for the state as a whole. However, this is not true for the sex–age–ethnic distribution of persons 40 years or older, since, as can be seen in his Table I, there is an under-representation of persons in the 40–49 age-group, for all of the sex–ethnic groups. In Hinds *et al.* (1980) age standardization of incidence rates was based on the world population standard. The percentage distribution of the Hawaiian population in 1970, by sex–age–ethnic group, and that of the world population standard are presented in Table I. It can be seen that the Hawaiian population was generally both younger than the population represented by the world standard, and with an overrepresentation of elderly persons in some sex–ethnic groups.

Another possible source of distortion relates to differences in past and current consumption patterns. Hinds *et al.* (1980) "assumed that current consumption rates for an ethnic–sex population reflect the past consumption rates", and claim that their "assumption should not cause distortion of exposure-incidence relationships if the *relative positions* of the 10 ethnic–sex groups, in respect of cigarette and alcohol use, have remained stable for the past 20 years". However, the slope estimate from the bivariate regression model used in their analysis will remain invariant only if past consumption rates can be expressed as a linear transformation of

TABLE I.—*Percent distribution of the population over 40 years of age in Hawaii (1970 & 1975) and world population standard*

| | | Ethnic–sex group | | | | | | | | | |
|---|---|---|---|---|---|---|---|---|---|---|---|
| | | Hawaiian | | Caucasian | | Chinese | | Filipino | | Japanese | |
| Age group | Population | Male | Female | Male | Female | Male | Female | Male | Female | Male | Female |
| 40–49 | H1970 | 46·00 | 45·79 | 47·16 | 42·38 | 35·58 | 35·65 | 23·34 | 50·43 | 38·98 | 42·43 |
| | H1975 | 37·43 | 38·02 | 38·08 | 35·80 | 25·00 | 29·92 | 22·52 | 48·31 | 27·69 | 33·53 |
| | World | 37·50 | 37·50 | 37·50 | 37·50 | 37·50 | 37·50 | 37·50 | 37·50 | 37·50 | 37·50 |
| 50–59 | H1970 | 31·10 | 29·54 | 27·80 | 28·39 | 26·39 | 26·70 | 26·57 | 20·82 | 32·32 | 29·85 |
| | H1975 | 34·22 | 33·33 | 30·96 | 29·91 | 33·97 | 33·86 | 21·40 | 31·46 | 39·72 | 37·87 |
| | World | 28·13 | 28·13 | 28·13 | 28·13 | 28·13 | 28·13 | 28·13 | 28·13 | 28·13 | 28·13 |
| 60–69 | H1970 | 13·98 | 15·89 | 15·00 | 14·32 | 21·56 | 21·54 | 35·36 | 18·21 | 17·30 | 13·92 |
| | H1975 | 19·79 | 22·92 | 18·89 | 20·84 | 23·08 | 22·83 | 38·29 | 13·11 | 19·91 | 17·16 |
| | World | 21·87 | 21·87 | 21·87 | 21·87 | 21·87 | 21·87 | 21·87 | 21·87 | 21·87 | 21·87 |
| 70+ | H1970 | 8·91 | 8·79 | 10·04 | 14·91 | 16·48 | 16·12 | 14·74 | 10·55 | 11·41 | 13·80 |
| | H1975 | 8·56 | 5·73 | 12·07 | 13·45 | 17·95 | 13·39 | 17·79 | 7·12 | 12·67 | 11·44 |
| | World | 12·50 | 12·50 | 12·50 | 12·50 | 12·50 | 12·50 | 12·50 | 12·50 | 12·50 | 12·50 |

Sources: For 1970, Rellahan *et al.* (1975).
For 1975, Kolonel (1979).
For world population, Doll & Cook (1967).

current rates. Preserving relative positions alone is not sufficient guarantee that the slope would not be affected.

A third source of distortion comes from differences in the target population of the survey and the population basis for the tumour registry. Usually, the target population for surveys like the one from which consumption habits are obtained is the civilian, non-institutionalized population. On the other hand, among the hospitals included in the Hawaiian Tumor Registry are military and state mental hospitals, as well as extended care facilities (Shambaugh, personal communication, 1981). It would be expected that consumption patterns of users of those resources differ significantly from patterns of persons represented in the survey.

Because the authors wished to assess separately the effects of smoking and type of alcoholic beverage (beer, spirits and wine) on the incidence rates, they performed an adjustment procedure on the data from the sample survey. Analysis of covariance was used for that purpose. Estimates of mean consumption for each of the 4 exposure variables were obtained and adjusted to a given level of consumption for the other 3 variables and age. Several observations should be made about the adjustment procedures. First, a linear relationship between the consumption variables being adjusted and the other 4 factors is assumed. Furthermore, the simplest form of covariance adjustment, which the authors used, assumes that the relationship between the variables is the same for each sex–ethnic group; *i.e.* the coefficients associated with the variables for which adjustment is being made are assumed to be the same for every group. The authors do not present any argument to support these assumptions. In fact, previous data (Kolonel, 1979) suggest that they are not valid, particularly when patterns of consumption among Chinese, Filipino and Japanese women are compared with the rest of the sex–ethnic groups.

Second, the adjustment of age-consumption data introduces distortions in the relationship between age-adjusted incidence rates and their associated consumption indicator values in the subsequent regression analyses on which the authors' conclusions are based. Thus, for example, the beer-consumption variables used in the regression analyses for beer was the mean consumption that would have been expected for groups all of which, on the average, consumed the same amount of wine, spirits and tobacco and had the same age. The values for these latter amounts are usually chosen to be the average amounts observed in the entire sample, because under proper conditions the adjusted consumption estimates will have the smallest errors at these levels. This procedure must be questioned (1) because it adjusts the independent variable (consumption) without a corresponding adjustment in the dependent variable (incidence), thereby introducing a bias of unknown magnitude, and (2) because of the arbitrary choice of levels of the variables for which adjustments are being made. It is most unlikely, *a priori*, that the true joint effect of these 4 variables on incidence is so simple and uncomplicated that it would remain invariant as those levels are changed, either as a group or relative to one another. An additional point about the age adjustment in the sample survey data is worth noting. The authors stated that the incidence rates were adjusted to the world population standard. The age adjustment in the sample survey data, if performed by standard covariance methods, would have adjusted to the observed average in the sample, which is most likely to be different from the corresponding age in the world cohort. The probability exists, therefore, that the dependent and independent variables in the regression analyses are not comparable with respect to age.

The analysis of exposure-incidence relationships relies on bivariate linear regression. The age-adjusted incidence rates were linearly regressed on the covariance-adjusted consumption indicators, and those regression coefficients which were statistically different from zero at the 5% probability level were noted. In the authors' analysis, the choice of the world population to standardize incidence rates was arbitrary, in the sense that it bore no relationship to the ages used for covariance adjustment of the independent variable. Different results may thus be obtained, depending on the choice of standard population. To study the effect of choice of standard, we replicated the analysis made by the authors, using different populations as standards.

Five-year incidence rates for the same sex–ethnic groups were obtained for the period 1973–77 (Young *et al.*, 1981). From scatter diagrams presented by Hinds *et al.* (1980) we were able to calculate the independent variables used in their analysis of consumption of beer and spirits. Although the values are not exactly the same, we were able to verify that they had the same mean and variance as those used by the authors. We repeated the analysis only for the 12 sites based on 1972–76 incidence rates. Our slopes, $R^2$ values and significance results were consistent with those obtained by the authors. We then calculated age-standardized rates using the 1973–77 age-specific rates, taking 3 populations as standard (World, European and African, Doll & Cook, 1967) and regressed them on the beer and spirit consumption adjusted means. Several discrepant results were obtained (Table II). However, the most disturbing inconsistency was unexpected. Different conclusions were obtained about the significance of the relationship between cancer of the kidney or lymphoma and beer consumption, and cancer of the brain and spirit consumption, depending on whether the 1972–76 or the 1973–77 data were used.

Much attention is being called at present to the undesirable characteristics of classical regression analysis and routine age-standardization in the interpretation of data for the identification of aetiological clues (Hickey *et al.*, 1980). It is well known that the classical least-squares estimator is very sensitive to extreme points. The inclusion in the Hinds *et al.* (1980) article of scatter diagrams is intended presumably to assess the effects of outliers on their statistical conclusions. However, the authors do not explain the criteria which were used to define points as outliers in some cases and not in others. For example,

TABLE II.—*Results of regression analyses*

| Site | World ('72–'76) | | World ('73–'77) | | European ('73–'77) | | African ('73–'77) | |
|---|---|---|---|---|---|---|---|---|
| | Beer | Spirits | Beer | Spirits | Beer | Spirits | Beer | Spirits |
| Oesophagus | * | | * | | * | | * | |
| Stomach | * | | * | | * | | * | |
| Colon | | | | | | | | |
| Rectum | | | | | | | | |
| Liver/biliary | | | * | | | | | |
| Pancreas | * | | * | | * | | * | |
| Lung | * | | * | | * | | * | |
| Kidney | * | | | | | | * | |
| Bladder | | * | | * | * | * | | * |
| Brain | | * | | | | | | |
| Lymphoma | | | * | | * | | * | |
| Leukaemia | * | | * | | * | | * | |

* Sites for which Ho: $\beta = 0$ is rejected at 5% level.

consideration is given as an outlying value to the incidence rate of leukaemia among Hawaiian males when beer consumption is being examined as a risk factor. This rate is about 1·6 times the largest incidence rate in the rest of the groups. For oesophagus that ratio is 2·6, but apparently it is not regarded as an outlier. They also consider the effect of excluding Caucasian males when spirit consumption is compared with incidence rates for cancer of the larynx, bladder and brain. However, no mention is made about the implications of exclusion of that sex–ethnic group when cancer of the pharynx is being analysed. For this site, the ratio of the largest to the second largest rate is greater than the corresponding ratios for larynx and brain. In fact, if Caucasian males are excluded, no significant relationship is found for any site. The proper question is not whether to exclude a given data-point. Instead, one should establish the implications of such actions and use alternative statistical techniques which are not as sensitive to these characteristics of data sets and which avoid the adoption of a given functional relationship involving an interpolation between two widely separated clusters of points.

Three final observations should be made about their regression analysis. First, it was based on the assumption of homoscedastic age-adjusted rates. The authors disregarded the fact that independent estimates of the standard errors can be obtained (Chiang, 1961) and incorporated as approximate weights in the regression analysis. Our calculations clearly show that the assumption of equal variances is not tenable. In fact, some of the rates have large coefficients of variation, suggesting that these rates should have been based on a longer period of time. Second, the significance tests are based on the assumption that the errors are normally distributed. It is generally agreed that this assumption is questionable for variables such as those considered by the authors. Finally, the use of the estimated regression lines to predict the percentage increase in cancer incidence rates due to a doubling of the population mean consumption of cigarettes and beer was uninformative. The percentage increases as calculated in their Table V depend on which baseline value is chosen. The authors chose 10 pack-years and 15 oz per week for cigarette and beer consumption respectively. Had they chosen different baselines, the percentage would have been different. The results they present are therefore arbitrary and not informative.

It has been shown that the application of well known statistical methods can lead to incorrect inferences when the assumptions on which the methods are based are not satisfied. In particular, the original analysis by Hinds *et al.* (1980) included inappropriate use of regression methods for adjustment, unweighted linear regression for heteroscedastic variables with non-linear relationships to consumption, omission of important interactions, and improper standardization of incidence rates. Although some of the statistical shortcomings in the analysis by Hinds *et al.* (1980) could have been avoided by using, for example, nonparametric techniques or more robust estimation methods on age-specific incidence rates and consumption indicators, the characteristics of the data set they use are such as to preclude appropriate epidemiological analysis.

This research was supported by a grant from the United States Brewers Association.

### REFERENCES

CHIANG, C. L. (1961) Standard error of the age-adjusted death rate. *Vital Statistics—Special Report, Selected Studies*, No. 9, Washington; USDHEW.

DOLL, R. & COOK, P. (1967) Summarizing indices for comparison of cancer incidence data. *Int. J. Cancer*, **2**, 269

HICKEY, R. J., CLELLAND, R. C. & CLELLAND, A. B. (1980) Epidemiological studies of chronic disease: Maladjustment of observed mortality rates. *Am. J. Public Hlth*, **70**, 142.

HINDS, M. W., KOLONEL, L. N., LEE, J. & HIROHATA, T. (1980) Associations between cancer incidence and alcohol/cigarette consumption among five ethnic groups in Hawaii. *Br. J. Cancer*, **41**, 929.

KOLONEL, L. (1979) Smoking and drinking patterns among different ethnic groups in Hawaii. *Natl Cancer Inst. Monogr.*, **53**, 81.

RELLAHAN, W, RASHAD, M N., BURCH, T. A. & OKINAGA, N. (1975) *Cancer Patterns in Hawaii*, 1960–1964 *and* 1968–1972. The Cancer Center of Hawaii and Hawaii State Department of Health.

YOUNG, J. L., JR, PERCY, C. L. & ASIRE, A. J. (1981) Surveillance epidemiology and end results: Cancer incidence and mortality 1973–77. *Natl Cancer Inst. Monog.*, **57**.